# Vitamin D-Binding Protein Clearance Ratio Is Significantly Associated with Glycemic Status and Diabetes Complications in a Predominantly Vitamin D-Deficient Population

**DOI:** 10.1155/2018/6239158

**Published:** 2018-05-20

**Authors:** Nabila A. Abdella, Olusegun A. Mojiminiyi

**Affiliations:** ^1^Department of Medicine, Faculty of Medicine, Kuwait University, Kuwait City, Kuwait; ^2^Department of Pathology, Faculty of Medicine, Kuwait University, Kuwait City, Kuwait

## Abstract

**Introduction:**

Studies have shown increased urine excretion of vitamin D-binding protein (VDBP) in patients with diabetic nephropathy (DN) resulting from postulated mechanisms linked to renal tubular damage. In this study, we evaluate the utility of VDBP clearance ratio as a novel determinant of glycemic status, DN, and other diabetes-associated complications.

**Methods:**

Levels of vitamin D, HbA1c, serum, urine concentrations of VDBP, and creatinine were measured in 309 subjects. The ratio of urine microalbumin to creatinine was determined to categorize subjects as normoalbuminuric (NAO), microalbuminuric (MIA), and macroalbuminuric (MAA). The VDBP clearance ratio was calculated.

**Results:**

Mean VDBP clearance ratios in NAO, MIA, and MAA were 0.7, 4, and 15, respectively. Significant positive correlations of VDBP clearance ratio were found with age, WC, SBP, DBP, TG, glucose, HbA1c, urine VDBP, urine microalbumin, and urine microalbumin/creatinine, and a significant negative correlation was found with the steady-state estimate of beta cell function (B%). Receiver operating curve (ROC) analyses of the use of VDBP clearance ratio for detection of albumin status shows a value of 0.81 for the area under the curve.

**Conclusions:**

The strong associations of VDBP clearance ratio with glycemic control and diabetes-associated complications suggest that this index could play a wider role in detection and/or pathogenesis and complications of diabetes.

## 1. Introduction

There is a worldwide deficiency of vitamin D that not only consequently leads to bone diseases but is also associated with cancer, diabetes, and heart disease. There is increasing evidence that supports the significant association of vitamin D deficiency (VDD) with the prevalence of metabolic syndrome and subsequent development of type 2 diabetes mellitus (type 2 DM) [1–3]. The main carrier of vitamin D in the serum is the low-molecular weight (58 kDa) glycoprotein vitamin D-binding protein (VDBP,) which significantly predicts the bioavailability of active levels of 25 (OH) vitamin D (25 (OH)D) in the bloodstream [4]. The VDBP/25 (OH) D complex formation, its filtration, and reabsorption through receptor-mediated uptake in proximal renal tubular cells are vital for the retrieval and activation of vitamin D. This reabsorption by endocytosis reduces VDBP excretion to minimal amounts. Therefore, tubular interstitial damage in patients with severe kidney disease would affect levels of urinary VDBP (uVDBP). Studies have reported increased concentrations of uVDBP among patients with chronic kidney disease [5, 6] that corresponded to increasing severity of kidney damage [6].

Diabetic nephropathy (DN) is one of the long-term complications of type 2 DM that leads to morbidity and mortality among individuals with diabetes. The prevalence of VDD and associated multiple microvascular complications in subjects with diabetes was significantly higher compared to subjects without diabetes [7]. Studies have also demonstrated a prominent relation between VDD/vitamin D insufficiency (VI) and DN [8].

Before the progression to renal dysfunction and end-stage renal disease, it is of clinical value to identify early markers that could be used for disease detection. Detection of microalbuminuria is used as a risk marker for early-stage identification of DN, but it is known to have low sensitivity and specificity [9]. Recent studies have demonstrated increased levels of VDBP excretion in patients with DN, and the resultant renal tubular dysfunction has been associated with increased levels of urinary VDBP in patients with type 1 DM and type 2 DM [5, 10]. Furthermore, some studies have suggested that VDBP may have biological actions beyond its main function as a vitamin D carrier protein. These functions include transport of fatty acids, sequestration of g-actin, and modulation of immune and inflammatory responses [11]. However, the potential of VDBP as a noninvasive risk marker for early detection of DN has not been well established. We hypothesized that increased urine loss of VDBP would contribute to the risk of developing type 2 DM and diabetic complications. In the present study, we explore the usefulness of VDBP : creatinine clearance ratio as a novel index of diabetes and its complications in a predominantly VDD/VI population. This measurement would help understand and evaluate its clinical significance as an independent predictor in early diagnosis and progression of the disease.

## 2. Materials and Methods

### 2.1. Subjects and Clinical Features

309 (124 M, 185 F) first-degree relatives of type 2 diabetic subjects, recruited from primary care polyclinics, were screened for glycemic and vitamin D status. The subjects were aged 38–70 years and were not known to have type 2 diabetes. The study was approved by the Ethics Committees of the Faculty of Medicine, Kuwait University, and the Ministry of Health, Kuwait, and performed in accordance with the Declaration of Helsinki. All subjects gave informed voluntary consent to participate in the study.

Waist circumference (WC) and blood pressure were measured as described previously [12]. WC was measured half way between the xiphisternum and the umbilicus at the point corresponding to the maximal abdominal protuberance. Two consecutive measurements of systolic blood pressure (SBP) and diastolic blood pressure (DBP) were taken from each subject after at least 10 min of rest. Hypertension was defined as an average (three readings) of SBP ≥ 140 mmHg, an average DBP ≥ 90 mmHg and/or self-reported current history of treatment for hypertension [13]. In addition, all subjects were assessed clinically using neuropathy symptom score (NSS), and the peripheral nerve function was assessed by light touch perception score (monofilament) as previously described [14]. Symptoms of autonomic disturbance were recorded, and all study subjects had standard autonomic function tests using a computer-based system (Vagus 2100, Sigma, Medizine-Technik, Germany)—Valsalva maneuver, R-R interval variation during deep breathing, heart rate response to standing, blood pressure response to sustained handgrip, and pupilometry were performed. Subjects were classified as normal or as having autonomic neuropathy based on established international criteria [15, 16].

### 2.2. Laboratory Methods

#### 2.2.1. Sample Collection

Urine (first early morning) samples and fasting blood samples were collected in the morning. Blood samples were centrifuged at 3000 rpm for 10 min. Samples were analyzed fresh or were aliquoted and stored at −80°C until further analysis.

#### 2.2.2. Assays

As described previously [12], fasting serum insulin was determined on an automated analyzer, Beckman DXI 800 ACCESS (Beckman Corporation), using the paramagnetic particle chemiluminescence immunoassay method. Insulin resistance was calculated using the homeostasis model assessment (HOMA-IR) using the HOMA2 calculator (version 2.2.2) downloaded from https://www.dtu.ox.ac.uk/homacalculator/download.php (Diabetes Trials Unit, Oxford). The HOMA2 calculator also gives estimates of steady-state beta cell function (B%) and insulin sensitivity (S%).

Fasting plasma glucose (FPG), alanine aminotransferase (ALT) activity, alkaline phosphatase (ALP), corrected calcium, phosphate, albumin, creatinine, total cholesterol (TC), triglycerides (TG), and high-density lipoprotein cholesterol (HDL-cholesterol) were analyzed on an automated analyzer (Beckman DXC 800, Beckman Corporation) [12]. The low-density lipoprotein cholesterol (LDL-cholesterol) was calculated using the Friedewald formula [17]. The formula is valid as long as TG ≤ 4.5 mmol/L.

HbA1c levels were measured using high-performance liquid chromatography on TOSOH G8 analyser (Tosoh Corporation, Tokyo, Japan) [12]. Fasting plasma glucose (FPG) or HbA1c values based on ADA diagnostic criteria [18] were used to categorize the subjects as follows: normal subjects: HbA1c < 5.7% or FPG < 5.6 mmol/L; subjects with prediabetes: HbA1c = 5.7%–6.4% or FPG = 5.6–6.9 mmol/L; and subjects with diabetes: HbA1c ≥ 6.5% or FPG ≥ 7.0 mmol/L.

Subjects were categorized based on the ratio of urine microalbumin to creatinine: normoalbuminuric for subjects with normal albuminuria (NAO, ratio < 30 mg/g), microalbuminuric for subjects with early DN (MIA, ratio 30–300 mg/g), and macroalbuminuric for subjects with severe DN (MAA, ratio > 300 mg/g).

Serum vitamin 25 (OH) D was measured by radioimmunoassay on Cobas e411. VDD was defined as vitamin D levels < 50 nmol/L, and VI was defined as vitamin D levels in the range of 50–75 nmol/L.

VDBP in plasma and urine samples were measured using a commercially available enzyme-linked immunoassay (ELISA) kit (Immunodiagnostik AG, Bensheim, Germany). The assay was performed according to manufacturer's instructions. The detection limit of the assay was estimated to be at 1.23 ng/mL. The intra-assay coefficient at the concentration of 24.2 mg/dL for 16 replicate determinations was 5% and interassay coefficient at the concentration of 19.3 mg/dL for 14 different assays was 12.7%.

VDBP clearance ratio was calculated as (UrineVDBP × SerumCreat)/(SerumVDBP × UrineCreat).

### 2.3. Statistical Methods

IBM SPSS 19.0 software (IBM) was used for statistical analysis. Data are presented as mean ± standard deviation (SD) unless otherwise specified. The Kolmogorov–Smirnov goodness-of-fit test was to test for normality of the data. Several variables (insulin, HOMA-IR, B%, and TG) that diverged significantly from normal distribution were log transformed when parametric tests were used. Comparisons between two groups were performed with the Mann–Whitney *U* test, and the Kruskal-Wallis analysis of variance was used to compare between more than two groups. The chi-square test was used to compare categorical variables. Univariate and multivariate analyses were used to compare study subjects grouped by vitamin D status, glycemic control, and degree of microalbuminuria. We performed receiver operating characteristic (ROC) analysis on the usefulness of VDBP clearance ratio for the diagnosis of VDD, microalbuminuria, and glycemic control. *P* < 0.05 was considered statistically significant for all analyses.

## 3. Results

### 3.1. Clinical and Biochemical Characteristics of Subjects Grouped by Microalbuminuria Status

Thirty-six (12%) subjects were found to have previously undiagnosed type 2 diabetes, 114 (37%) subjects had prediabetes, and 159 (51%) subjects were normoglycemic. The characteristics of subjects grouped by microalbuminuria status are shown in Table 1. VDD (<50 nmol/L) or insufficiency (VI) (50–75 nmol/L) was prevalent. 249 subjects were NAO, 48 were MIA, and 12 were MAA. 86 (28%) of the study subjects had peripheral sensory neuropathy and 29 (9%) had autonomic neuropathy. Urinary VDBP strongly increased with microalbuminuria status, but plasma VDBP levels remain unchanged. This correlated with significant elevation of VDBP clearance ratio in macroalbuminuric and microalbuminuric subjects when compared to normoalbuminuric subjects. Mean VDBP clearance ratios in NAO, MIA, and MAA were 0.7, 4, and 15, respectively.

### 3.2. Correlations of VDBP Clearance Ratio

The correlations of VDBP clearance among subjects are shown in Table 2. VDBP clearance ratio showed significant positive correlation with age (*r* = 0.28), WC (*r* = 0.18), SBP (*r* = 0.20), DBP (*r* = 0.26), TG (*r* = 0.19), glucose (*r* = 0.31), HbA1c (*r* = 0.35), urine VDBP (*r* = 0.95), urine microalbumin (*r* = 0.31), and urine microalbumin/creatinine (*r* = 0.44) and significant negative correlation with B% (*r* = −0.27).

### 3.3. Regression Analysis

The results of binary logistic regression are shown in Table 3. The VDBP clearance ratio displays significant independent association with microalbuminuria. The association remained significant even in the presence of confounding variables like age, WC, and HbA1c.

### 3.4. VDBP Clearance Ratio Associations

**Figure 1:**
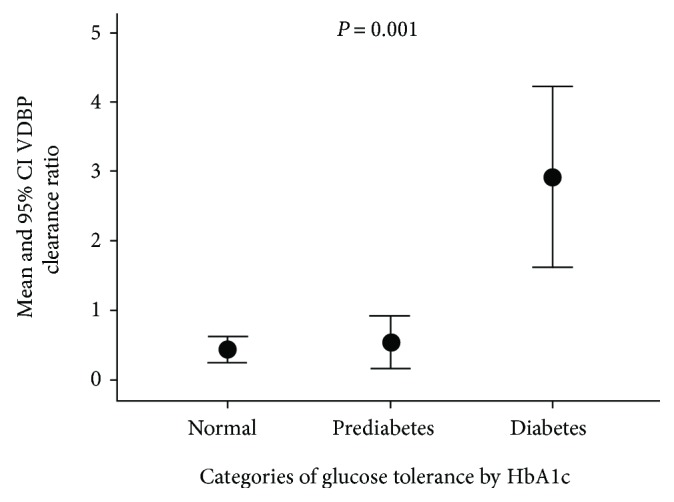


In Figure 1, subjects are categorized based on the glucose tolerance determined by HbA1c levels. The VDBP clearance ratio in subjects with diabetes showed significantly higher VDBP ratio when compared to those with prediabetes as well as in subjects with normal HbA1c levels.

**Figure 2:**
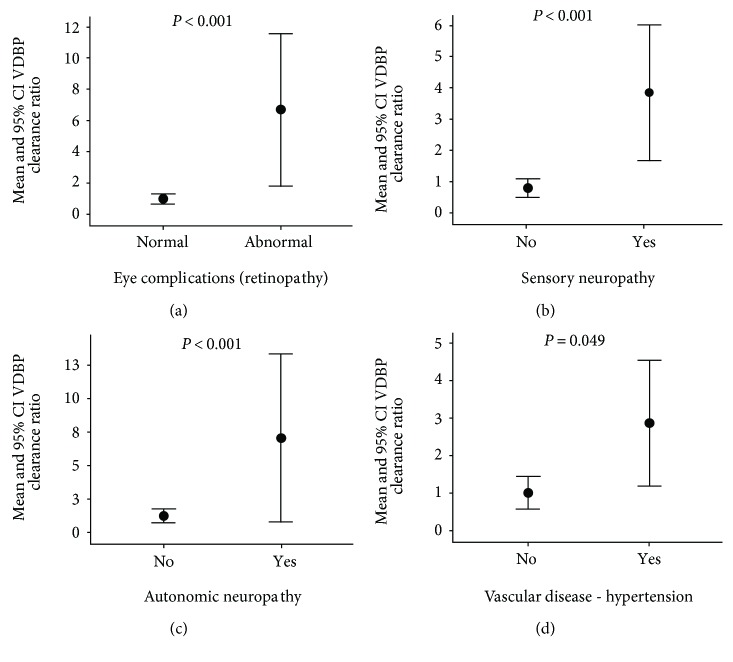


Figure 2 shows the association of VDBP clearance ratio with microvascular and macrovascular complications of diabetes. The VDBP ratio was higher in subjects with microvascular complications such as eye complications (Figure 2(a)), sensory neuropathy (Figure 2(b)), and autonomic neuropathy (Figure 2(c)) as well as macrovascular-associated risk factor like vascular hypertension (Figure 2(d)).

### 3.5. ROC Curve Analysis for Detection of Microalbuminuria Status

**Figure 3:**
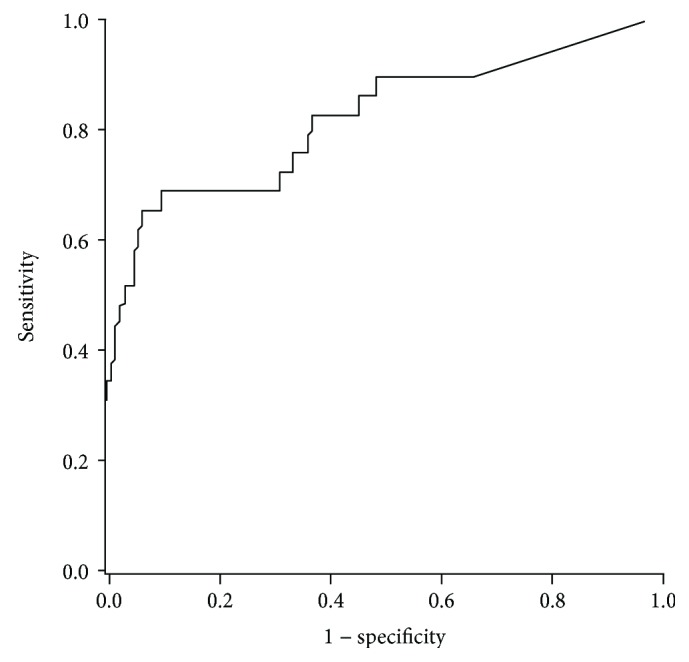


In Figure 3, ROC curve analyses for the use of VDBP clearance ratio to determine microalbuminuria status show that the area under the curve for VDBP clearance ratio is 0.81. At a cut-off point of 0.52, the sensitivity and specificity of VDBP clearance ratio for the detection of microalbuminuria are 83% and 67%, respectively.

## 4. Discussion

VDD is common among patients with chronic kidney disease including DN. Over the years, several factors have been suggested to contribute to reduced circulating concentrations of vitamin D from reduced exposure to sunlight and poor nutrition to increased excretion of uVDBP accompanied by proteinuria [19, 20]. Studies have shown that low vitamin D is a risk factor for type 2 DM development [2, 21] and that vitamin D/Ca supplementation could regulate glucose metabolism, thereby reducing risk [22]. In this study, we found a high prevalence of VDD and VI among subjects irrespective of the degree of microalbuminuria (Table 1). Similar findings were reported in a cross-sectional analysis among individuals with diabetes [8], which highlights the potential role of vitamin D in glucose metabolism and insulin sensitivity. VDBP polymorphisms and low levels of vitamin D have also been shown to be associated with insulin deficiency and development of type 2 DM [23, 24].

The increased excretion of urine VDBP has been demonstrated in various diseases [25–27] but only more recently shown in DN. In our study, we assessed vitamin D and its associated analytes with different stages of renal damage based on microalbuminuria status. Although strong positive elevation of urinary VDBP levels was observed, plasma VDBP and vitamin D levels remain unaffected among subjects with MIA/MAA when compared to those with NAO (Table 1). The vitamin D/VDBP ratio also remained the same across NAO, MIA, and MAA subjects suggesting that despite the urinary loss of VDBP, free and bioavailable vitamin D remains the same (Table 1).

Unlike other studies where urinary concentrations of VDBP was evaluated, we assessed the ratio of VDBP excreted in the urine taking into account its levels in the plasma, thereby providing a more accurate measure of filtered VDBP. Levels of VDBP clearance ratio in the group with albuminuria were increased to more than sixfold when compared to the group with normoalbuminuria (Table 1). The quantitative VDBP clearance ratio significantly and progressively increased with increasing degrees of microalbuminuria. Additionally, binary logistic regression analysis reveals the VDBP clearance ratio to be independently associated with microalbuminuria (Table 3). These findings suggest not only its positive prominent association of VDBP clearance ratio with disease progression of nephropathy but also its potential as an interesting candidate biomarker in early diagnosis. The substantial increase of VDBP clearance ratio among VDD/VI subjects was largely contributed by increased VDBP in excretion. In agreement with our findings, several earlier studies reported an increase in uVDBP loss with disease severity in diabetic nephropathy [5, 6]. Previous studies have consistently shown increased levels of uVDBP in subjects with diabetic nephropathy compared to those without [10, 28]. In one study, among several urinary proteins upregulated in diabetic nephropathy, VDBP demonstrated the highest with an 11-fold increase compared to the control group [29]. More recently, ROC studies among type 2 DM groups divided according to albumin : creatinine ratio have established optimum cut-off values of uVDBP to be between 550 and 553 ng/mg that correspond to >90% sensitivity and >83% specificity for early detection/diagnosis of diabetic nephropathy [10, 30]. Based on the VDBP clearance ratio diagnostic performance, our findings further validate its potential role as an early predictor of kidney disease (Figure 3).

Tharakilil et al., in their study in patients with type 1 DM, indicated that the presence of VDD/VI was linked to enhanced urinary loss of VDBP due to the mechanistic involvement of VDBD/25 (OH) complex in the proximal tubules for processes and activation of vitamin D [5]. In another study however antiproteinuric intervention substantially reduced urinary loss of VDBP, which did not associate significantly with vitamin D status. Due to the limited group sizes in their study, the inference of a lack of contribution of VDBP loss in urine to VDD is nonconclusive [31]. In a more recent report among patients with chronic kidney disease, the loss of VDBP in urine correlated with proteinuria but had no association with VDD and serum concentrations of VDBP [32]. Their results largely support our findings with VDBP clearance ratio, which showed no association with vitamin D status and plasma VDBP levels (Table 2).

There is evidence that supports the association of glycemic control with changes in serum and urine concentrations of VDBP in diabetes [33]. In our study, we show strong correlations of VDBP clearance ratio with metabolic factors associated with adverse type 2 DM control such as WC, BP, TG, and glucose levels (Table 2). The VDBP clearance ratio showed a significant stepwise increase with categories of glucose tolerance (Figure 1). These factors suggest that VDBP clearance ratio is dependent on glycemic control, which is a determinant of the onset and progression of DN. However, the fact that binary regression analyses showed that the association of VDBP clearance ratio with DN status is independent of HbA1c suggests that the mechanisms of VDBP clearance in DN and poor glycemic control may not necessarily be interlinked. Apart from DN, we also demonstrate increased VDBP in excretion to significantly predict other diabetes-associated complications like retinopathy, neuropathy, and hypertension (Figure 2). However, these findings may be due to the known co-occurrence of these complications with DN. To our knowledge, our findings are the first to demonstrate substantially increased VDBP clearance and loss with diabetic complications.

The exact mechanism of increased VDBP excretion is not clearly understood, but few reports have highlighted some factors. The endocytotic receptor pathway is involved in the reabsorption of filtered 25 (OH) D and VDBP in the glomerulus [34]. The enhanced excretion of VDBP as observed in our results and others may be linked to disease severity in DN as a result of renal tubular cell damage. Megalin is a multiligand endocytotic receptor involved in the tubular clearance and reabsorption of albumin and other proteins like VDBP, lipoproteins, and hormones in the proximal tubules. Cubulin is another endocytotic glycoprotein, which, in complex with megalin, participates in renal uptake of several ligands including vitamin D carrier proteins. Loss of function and shedding of megalin/cubulin in kidney disease lead to albuminuria and, in turn, could contribute to enhanced urinary excretion of VDBP [35]. Mirković et al. [6] in their study showed that the increased overall loss of VDBP in urine could be linked to tubular interstitial damage that can be modulated by antiproteinuric treatment. These findings further support the fact that VDBP excretion and/or clearance could be candidate biomarkers in proteinuric chronic kidney disease.

The main limitation of this study is the cross-sectional nature of the study that cannot precisely determine the mechanistic role of VDBP clearance ratio in diabetes and DN. More studies are required to understand the mechanistic role of VDBP and its clearance in pathogenesis and progression of DN as well as in other diabetic complications. This would largely benefit early identification of those patients with diabetes at risk of developing complications as well as improve management of the disease. Increased VDBP loss did not account for VDD among our study subjects, and further studies are required to confirm its contribution to vitamin D status and response to treatment of VDD.

## 5. Conclusions

VDBP clearance ratio is significantly associated with glycemic control and diabetes-associated complications. This index could play a role in the detection and/or pathogenesis of diabetic complications.

## Figures and Tables

**Table 1:** Characteristics of study participants grouped by microalbuminuria status.

Variables	Microalbuminuria status			*P*
	Normoalbuminuric	Microalbuminuric	Macroalbuminuric	
*n*	249	48	12	
Age (years)	42.3 ± 17.6	52.5 ± 17.1	60.0 ± 10.8	<0.001
WC (cm)	99 ± 15	103 ± 13	109 ± 15	<0.001
SBP (mmHg)	127 ± 17	137 ± 20	148 ± 22	<0.001
DBP (mmHg)	78 ± 13	80 ± 14	84 ± 13	0.013
HDL-C (mmol/L)	1.16 ± 0.31	1.16 ± 0.26	1.14 ± 0.32	0.920
LDL-C (mmol/L)	2.84 ± 0.90	2.64 ± 0.85	2.93 ± 0.94	0.658
ALP (IU/L)	76.65 ± 36.06	84.31 ± 49.74	89.0 ± 39.75	0.016
ALT (IU/L)	24.98 ± 16.0	23.31 ± 10.96	24.29 ± 10.99	0.783
TG (mmol/L)	1.41 ± 1.10	1.73 ± 1.02	1.80 ± 1.01	0.036
TC (mmol/L)	4.61 ± 1.02	4.59 ± 1.05	4.79 ± 1.05	0.708
Glucose (mmol/L)	7.11 ± 3.43	10.05 ± 4.39	11.41 ± 4.43	<0.001
HbA1c (%) (mmol/mol)	6.9 ± 2.0 (52)	8.7 ± 2.4 (72)	9.8 ± 2.4 (84)	<0.001
Serum creatinine (*μ*mol/L)	61.60 ± 16.48	69.83 ± 33.39	86.38 ± 42.78	<0.001
Corrected calcium (mmol/L)	2.36 ± 0.15	2.39 ± 0.15	2.39 ± 0.09	0.001
Phosphate (mmol/L)	1.24 ± 0.20	1.26 ± 0.20	1.22 ± 0.16	0.845
Vitamin D (nmol/L)	44.55 ± 32.73	45.45 ± 33.27	57.85 ± 58.38	0.633
Plasma VDBP (*μ*g/mL)	372.84 ± 112.67	373.52 ± 93.14	339.26 ± 60.07	0.378
Vitamin D/albumin	1.12 ± 0.85	1.19 ± 0.93	1.58 ± 1.59	0.655
Vitamin D/VDBP	<0.001	<0.001	<0.001	0.364
VDBP clearance ratio	0.66 ± 1.02	4.01 ± 6.28	14.99 ± 10.24	<0.001
Urine VDBP (ng/mL)	53.96 ± 113.70	203.39 ± 297.02	541.48 ± 466.83	0.002
Urine microalbumin/creatinine (mg/g)	7.88 ± 5.90	83.54 ± 61.14	1064.79 ± 894.27	<0.001

WC: waist circumference; SBP: systolic blood pressure; DBP: diastolic blood pressure; HDL-C: high-density lipoprotein; LDL-C: low-density lipoprotein; ALP: alkaline phosphate; ALT: alanine aminotransferase; TG: triglyceride; TC: total cholesterol; HbA1c: hemoglobin A1c; plasma VDBP: plasma vitamin D-binding protein; vitamin D/VDBP: vitamin D to vitamin D-binding protein ratio; VDBP clearance ratio: vitamin D-binding protein clearance ratio; urine VDBP: urine vitamin D-binding protein.

**Table 2:** Spearman rank correlations of VDBP clearance ratio with metabolic and other variables.

Variable	Correlation coefficient	*P*
Age (years)	0.28	0.001
WC (cm)	0.18	0.028
SBP (mmHg)	0.20	0.015
DBP (mmHg)	0.26	0.001
LDL-C (mmol/L)	−0.04	0.623
HDL-C (mmol/L)	−0.14	0.080
ALP (IU/L)	0.05	0.527
ALT (IU/L)	0.08	0.352
TG (mmol/L)	0.19	0.021
TC (mmol/L)	−0.01	0.908
Serum creatinine (*μ*mol/L)	0.10	0.203
Total protein (g/L)	0.07	0.386
Albumin (g/L)	−0.14	0.093
Corrected calcium (mmol/L)	0.09	0.262
B%	−0.27	0.002
S%	−0.07	0.426
HOMA-IR	−0.01	0.948
Insulin (*μ*IU/mL)	0.06	0.486
Glucose (mmol/L)	0.31	<0.001
HbA1c (%)	0.35	<0.001
Vitamin D (nmol/L)	0.02	0.794
Plasma VDBP (mg/mL)	−0.13	0.128
Vitamin D/albumin	0.03	0.695
Vitamin D/VDBP	0.08	0.333
Urine VDBP (ng/mL)	0.95	<0.001
Urine microalbumin (mg/L)	0.31	<0.001
Urine microalbumin/creatinine (mg/g)	0.44	<0.001

WC: waist circumference; SBP: systolic blood pressure; DBP: diastolic blood pressure; LDL-C: low-density lipoprotein; HDL-C: high-density lipoprotein; ALP: alkaline phosphate; ALT: alanine aminotransferase; TG: triglyceride; TC: total cholesterol; B%: beta cell function; S%: insulin sensitivity; HOMA-IR: homeostasis model assessment of insulin resistance; HbA1c: hemoglobin A1c; plasma VDBP: plasma vitamin D-binding protein; vitamin D/VDBP: vitamin D-to-vitamin D-binding protein ratio; urine VDBP: urine vitamin D-binding protein.

**Table 3:** Binary logistic regression of the association of VDBP clearance ratio with microalbuminuria.

VDBP clearance ratio	OR	95% CI	*P*
Crude	1.999	1.472–2.715	<0.001
With inclusion of age	1.821	1.356–2.446	<0.001
With inclusion of age and WC	1.800	1.346–2.408	<0.001
With inclusion of age, WC, and HbA1c	1.607	1.199–2.152	0.001

VDBP clearance ratio: vitamin D-binding protein clearance ratio; WC: waist circumference; HbA1c: hemoglobin A1c.

## Data Availability

The authors have discussed the issue of data availability with the local Ethics Committees and the funding body, Kuwait Foundation for the Advancement of Sciences (KFAS). The authors are unable to share the data based on the following: (1) Ethics Committee approval: The local Ethics Committees expressly forbid data sharing based on issues related to patient confidentiality, privacy, and related concerns. Such permission should have been included in the original ethics permission and cannot be granted retrospectively. (2) KFAS does not allow public data sharing for its funded projects. In addition to the above reasons, the authors have concerns with regard to the copyright of data shared publicly. The corresponding author and principal investigator are willing to consider access to specific aspects of the data on a case-by-case basis if there is sufficient evidence that the data are needed for collaborative publications that would include or acknowledge all the authors in the current submission. Please note that such permission will ultimately depend on approval by the local Ethics Committees and KFAS.
