# Corrigendum to “Vitamin D-Binding Protein Clearance Ratio Is Significantly Associated with Glycemic Status and Diabetes Complications in a Predominantly Vitamin D-Deficient Population”

**DOI:** 10.1155/2018/3016723

**Published:** 2018-10-22

**Authors:** Nabila A. Abdella, Olusegun A. Mojiminiyi

**Affiliations:** ^1^Department of Medicine, Faculty of Medicine, Kuwait University, Kuwait City, Kuwait; ^2^Department of Pathology, Faculty of Medicine, Kuwait University, Kuwait City, Kuwait

In the article titled “Vitamin D-Binding Protein Clearance Ratio Is Significantly Associated with Glycemic Status and Diabetes Complications in a Predominantly Vitamin D-Deficient Population” [1], the mention of “radioimmunoassay” in the Materials and Methods was incorrect, where the sentence “Serum vitamin 25 (OH) D was measured by radioimmunoassay on Cobas e411.” should be corrected to “Serum vitamin 25 (OH) D was measured by electrochemiluminescence immunoassay on Cobas e411.”
